# Restricted Crystalloid Fluid Therapy during Orthotopic Liver Transplant Surgery and its Effect on Respiratory and Renal Insufficiency in the Early Post-operative Period: A Randomized Clinical Trial

**Published:** 2014-08-01

**Authors:** M. A. Sahmeddini, F. Janatmakan, M. B. Khosravi, S. Ghaffaripour, M. H. Eghbal, S. Nickeghbalian, S. A. Malek-Hosseini

**Affiliations:** 1 Shiraz Anesthesiology and Critical Care Research Center, Shiraz University of Medical Sciences, Shiraz, Iran; 2 Ahvaz Jundishapur University of Medical Sciences, Ahvaz, Iran; 3 *Transplant Research Center, Shiraz University of Medical Sciences, Shiraz, Iran*

**Keywords:** Liver transplantation, Acute renal insufficiency, Respiratory insufficiency, Postoperative period, Mortality, Morbidity

## Abstract

Background: Respiratory and renal insufficiencies are common dysfunctions during post-liver transplantation period that increase post-operative mortality and morbidity rates. Intra-operative fluid therapy is an important factor associated with pulmonary and renal insufficiency.

Objective: To evaluate the relation between intra-operative fluid therapy and early renal and respiratory insufficiency after liver transplantation.

Methods: In this randomized clinical study, 67 adult patients with end-stage liver disease who underwent orthotopic deceased donor liver transplantation were randomly allocated into two groups. The restricted fluid group, which received a controlled fluid administration of normal saline, 5 mL/kg/hr during anesthesia, and non-restricted fluid group received a controlled infusion of normal saline 10 mL/kg/hr during anesthesia. Early post-operative respiratory and renal insufficiency in both groups were assessed. The patients were monitored during the three stages of liver transplantation for their hemodynamic indices. The trial is registered with the Iranian Randomized Clinical Trial Registry, number IRCT2013101811662N4.

Results: The baseline demographic and clinical characteristics were similar in both studied groups. The prevalence of respiratory insufficiency in the non-restricted fluid group (15%) significantly (p=0.01) higher than that in the restricted fluid group (0%). The post-operative mean±SD serum creatinine was 1.0±0.1 mg/dL in the non-restricted fluid group and 1.1±0.2 in the restricted fluid group (p=0.43). No patients in the studied groups required post-operative continuous renal replacement therapy.

Conclusions: Restricted crystalloid fluid administration during orthotropic liver transplantation though decreased post-operative chance of pulmonary insufficiency, did not increase renal dysfunction.

## INTRODUCTION

During the past few decades, organ transplantation has evolved from an experimental treatment to a standard treatment option for patients with end-stage organ diseases. This is mainly due to innovations in surgical techniques, peri-operative care, and improvements in immunosuppressive regimens [1, 2]. 

End-stage liver disease (ESLD) is associated with low systemic vascular resistance (SVR) caused by peripheral vasodilatation [3-5]. When orthotopic liver transplantation is undertaken during general anesthesia (GA), this vasodilatation is aggravated by the natural vasodilating property of anesthetic agents [6]. The consequential intense decrease in SVR leads to significant hypotension requiring substantial volumes of intravenous (IV) fluid administration to preserve a stable hemodynamic status. The excessive IV fluid administered ultimately translocates from the intravascular compartment to the lungs in the post-operative period [7, 8]. Furthermore, development of renal dysfunction is facilitated by circulatory changes such as splanchnic vasodilation, which are usually seen in ESLD patients with portal hypertension [9]. This vasodilation causes ineffective arterial underfilling with subsequent constriction of the renal arteries [10, 11].

Respiratory and renal insufficiencies are common after liver transplantation and could lead to post-operative mortality and morbidity [12]. However, these complications are related to intra-operative fluid therapy [13, 14]. Some studies showed that constraining peri-operative fluid therapy decreases the incidence of early respiratory insufficiency [15]; another study, on the other hand, showed that patients with ESLD have a fragile renal system so it is important to avoid hypovolemic stress on their kidneys during liver transplantation [16]. Restricted intra-operative fluid therapy may cause more renal damage so attention should be paid to fluid therapy during the intra-operative period [15]. Furthermore, increasing the splanchnic arterial tone with vasoconstrictors may reverse renal dysfunction [17].

The present study was conducted to investigate whether restricted crystalloid fluid therapy during anesthesia for orthotopic liver transplantation (OLT) decreases early post-operative respiratory and renal insufficiencies.

## PATIENTS AND METHODS

This study was as a single-center, non-blinded, non-placebo controlled, parallel groups with balance randomization clinical trial that conducted in Iran. The trial is registered with the Iranian Randomized Clinical Trial Registry, number IRCT2013101811662N4. From February to November, 2010, 67 adult patients with ESLD who underwent orthotopic deceased donor liver transplantation in Shiraz organ transplantation center enrolled in this study. Patients with a history of asthma, chronic obstructive lung disease, hepatopulmonary syndrome, hepatopulmonary hypertension, hepatorenal syndrome, and renal failure were excluded from the study.

After obtaining approval from our institutional ethics committee and taking informed written consent from participants, using block randomization, patients were randomly categorized into two treatment arms to receive either “restricted crystalloid fluid” (5 mL/kg/hr) or “non-restricted crystalloid fluid” (10 mL/kg/hr) by a nurse who had no role in the clinical management of the patients.

The induction of anesthesia was accomplished with thiopental (5 mg/kg), fentanyl (2 µg/kg), and midazolam (0.03 mg/kg). Pancuronium (0.1 mg/kg) or atracurium (0.6 mg/kg) was used for neuromuscular blockade. Ventilation was maintained with an air-oxygen mixture plus isoflurane. Calcium gluconate was used to correct for low calcium (Ca^2+^) level. Cardiovascular function was monitored using an electrocardiogram, a radial artery catheter, and measurement of central venous pressure via the right internal jugular vein. Cardiac output was measured by NICO system (Novametrix-Medical System Inc, for measuring non-invasive Co); therefore, NaHCO_3_ was not used for correction of acidosis in this study.

Transplantation of the graft was performed using the piggy-back technique. The operation consisted of three phases: 1) the hepatectomy phase, 2) anhepatic phase, and 3) neohepatic phase. The total ischemic time was defined as the period from the aortic cross-clamping and perfusion with preservation solution in donor to the completion of the anastomosis with portal reperfusion.

In the restricted crystalloid fluid group, 5% albumin, fresh frozen plasma and packed cells were administrated to maintain the central venous pressure at 8–10 cm H_2_O, the hematocrits at almost 30%, and as soon as the mean arterial pressure (MAP) dropped to <60 mm Hg. Patients in this group were given norepinephrine as vasopressor with an initial dose of 0.05 µg/kg/min; the dosage was increased until the MAP was maintained at ≥60 mm Hg. If drainage of ascitic fluid in patients of this group caused hypotension, the patients were given norepinephrine and the drained ascitic fluid was not replaced with crystalloids. 

In the non-restricted crystalloid fluid group, normal saline 10 mL/kg/hr, and packed cells were administrated to maintain the central venous pressure at >10 cm H_2_O, and the hematocrit at almost 30% to maintain the MAP at almost 60 mm Hg; the patients whose MAP remained <60 mm Hg with fluid therapy, were given norepinephrine with an initial dose of 0.05 µg/kg/min; the dosage was increased until the MAP was maintained at ≥60 mm Hg. If patients in this group had ascites, the drained ascitic fluid was replaced with normal saline and 25% albumin to maintain the blood pressure.

The primary outcome was acute respiratory and acute renal insufficiency. Acute respiratory insufficiency was defined as p_a_O_2_ <60 mm Hg or p_a_CO_2_ >45 mm Hg measured on the 1^st^ day, 2^nd^ day, and 3^rd^ day after the operation. According to these criteria, reintubation and ventilatory support was started for patients. 

Acute renal insufficiency was defined as a rise in serum creatinine (in the absence of primary graft failure) that was measured by Jaffe/kinetic method on the 1^st^, 2^nd^, and 3^rd^ post-operative days.

The secondary outcome was hemodynamic indices—MAP, heart rate, central venous pressure, and cardiac output. MAP and central venous pressure were measured through arterial line and the double lumen central venous access line, respectively. Heart rate and cardiac output were measured by electrocardiogram and NICO system, respectively. 

**Statistical Analysis**

Assuming a study power of 80%, type I error of 5%, an effect size of 10 mEq/L in base excess, an expected SD of 14.5 mEq/L in both study arms, Power SSC ver 2.00, revealed a minimum sample size of 34 patients in each study arm (a total of 68 patients).

Data were analyzed by SPSS ver 14.0 for Mac OS X. Discrete variables were compared using the χ^2^ test or Fisher’s exact test when necessary. Continuous variables were compared by one-way analysis of variance (ANOVA). All values were presented as mean±SD. A p value <0.05 was considered statistically significant.

## RESULTS

Of 81 patients who were candidate for OLT from February 2010 to November 2010, 14 were excluded from the study (five with hepatorenal syndrome, one had hepatopulmonary syndrome, in six surgical technique was not piggy back, and two had chronic obstructive lung disease). Of the remaining 67 patients who fulfilled the inclusion criteria, 34 were allocated to restricted crystalloid fluid group, and 33 were allocated to non-restricted crystalloid fluid.

The two groups were similar in terms of baseline demographic characteristics of the donors and the recipients and graft factors (Tables 1 and 2). 

**Table 1:** Demographic characteristics in the recipients of studied groups. Values are presented as mean**±**SD or number of cases.

**Variable**	**Restricted Crystalloid fluid group (n=34)**	**Non-restricted Crystalloid fluid group (n=33)**	**p value**
**Sex (M/F)**	20/14	23/10	0.07
**Age (yr)**	27.5±5.6	26.2±8.2	0.72
**MELD score** *	20.5±3.5	19.4±2.9	0.59
**Body Weight (kg)**	65.5±7.4	59.4±9.1	0.08
**Height (cm)**	162.5±9.9	169.6±7.6	0.8

* MELD: Model for end-stage liver disease

**Table 2:** Demographic characteristics in the donors of studied groups. Values are presented as mean**±**SD or number of cases.

**Variable**	**Restricted crystalloid fluid group (n=34)**	**Non-restricted crystalloid fluid group (n=33)**	**p value**
**Sex (M/F)**	30/4	28/5	0.08
**Donor age (yr)**	28.6±8.0	30.9±6.5	0.30
**Fatty change liver (%)**	11.3±8.1	13.2±6.8	0.60
**Graft weigh/recipient weight**	1.2±0.2	1.2±0.3	0.40
**Total ischemic time (Hr)**	9.5±1.8	10.9±1.5	0.20

Table 3 compares the hemodynamic variables between the two treatment groups at various stages of liver transplantation. During the hepatectomy phase, an-hepatic phase, and neo-hepatic phase in the restricted crystalloid fluid group the central venous pressure was significantly (p<0.05) lower than the non-restricted crystalloid fluid group. The MAP was significantly (p<0.05) higher in the restricted crystalloid fluid group than the non-restricted crystalloid fluid group during the hepatectomy phase, an-hepatic phase and neo-hepatic phase (Table 3). The mean±SD dose of norepinephrine given to patients in the restricted crystalloid fluid group was 0.25±0.09 µg/kg/min, not significantly (p=0.08) different from that given to those in non-restricted crystalloid fluid group 0.21±0.06 µg/kg/min. However, we started norepinephrine infusion in the restricted crystalloid fluid group earlier than in the non-restricted crystalloid fluid group to maintained the MAP at levels ≥60 mm Hg. No significant differences was observed between the two groups in terms of heart rate and cardiac output (Table 3).

**Table 3:** Hemodynamic parameters measured during three stages of the liver transplantation in the study groups. Values are mean±SD

**Variable**	**Restricted** **Crystalloid fluid group (n=34)**	**Non-restricted ** **Crystalloid fluid group (n=33)**	**p value**
**Baseline**			
MAP* (mm Hg)	70.6± 3.9	70.0±2.9	0.20
Heart rate (beats/min)	112.5±5.9	108.8±6.8	0.43
CVP† (mm Hg)	7.9±2.7	7.0±3.4	0.56
**Hepatectomy phase**			
MAP(mm Hg)	72.5±6.9	61.1±2.8	0.01
Hear rate (beats/min)	87.6± 3.7	90.8±1.9	0.03
CVP (mm Hg) ‡CO (L/min)	8.2±2.8 6.7±0.6	13.9±2.9 6.6±1.0	0.001 0.38
**An-hepatic phase**			
MAP (mmHg)	67.3±2.6	55.8±3.7	0.001
Hear rate (beats/min)	90.8±2.0	109.2 ±2.7	0.01
CVP (mm Hg) CO (L/min)	8.7±1.5 4.3±0.05	12.8±3.0 5.0±0.05	0.02 0.08
**Neo-hepatic phase**			
MAP (mm Hg)	75.78 ±3.01	61.1±1.1	0.001
Hear rate (beats/min)	84.65±1.98	81.9±3.1	0.24
CVP (mm Hg) CO (L/min)	8.51±1.02 5.43±0.78	15.0±2.1 5.0±0.8	0.01 0.71

* MAP: Mean arterial pressure

† CVP: Central venous pressure

‡ CO: Cardiac output

The were statistically significant difference between the study groups in the prevalence of endotracheal reintubation (p=0.01), mechanical ventilation (p=0.01), and duration of ICU stay (p=0.003) in post-operative period (Table 4). While intra-operative urine output was significantly (p<0.001) lower in the restricted crystalloid fluid group compare to non-restricted crystalloid (Table 4), there were no significant differences in the post-operative urine output (p=0.098) and the post-operative serum creatinine (p=0.43) between the two groups (Table 4). No patient required continuous renal replacement therapy due to renal insufficiency in post-operative period (Table 4).

**Table 4:** Comparison of pulmonary and renal end points of between the vasopressor and the control group. Values represent mean±SD or number (%) of patients

Parameter	**Restricted Crystalloid fluid group (n=34)**	**Non-restricted Crystalloid fluid group (n=33)**	**p value**
**Duration of ICU stay(d)**	1.9±0.5	2.7±0.4	0.003
**No. of patients needing endotracheal reintubation**	0 (0%)	5 (15%)	0.01
**No. of patients with pulmonary edema**	0 (0%)	5 (15%)	0.01
**Intra-operative urine output (mL)**	450.9±8.6	652.4±9.9	<0.001
**Post-operative urine output (mL)**	2505.6±50.1	2490.5±48.1	0.10
**Post-operative creatinine (mg/dL)**	1.1±0.2	1.0±0.1	0.43
**No. of patients needing post-operative CRRT** *	0 (0%)	0 (0%)	0.67

* CRRT: Continuous renal replacement therapy

## DISCUSSION

We found that if to maintain the MAP in patients undergoing OLT, less crystalloid fluid is administered during anesthesia, lesser post-operative respiratory insufficiency would occur.

ESLD is associated with low SVR, for which, sodium and water are retained by the kidneys, which in turn increases the amount of total body fluid [18, 19]. Splanchnic circulation is expanded because of portal hypertension; total blood volume increases but the relative amount of blood in the systemic circulation falls [20, 21]. In addition, because of this portal hypertension, the distribution of total body fluid is altered, protein-rich fluid moves into the body cavities and causes ascites and pleural effusion [22]. 

Based on a study by Jiang, *et al* [23], intra-operative and post-operative fluid therapy are important factors related to pulmonary complications and recovery. Ponnudurai, *et al* [24], showed that a titrated infusion of norepinephrine and controlled IV fluid administration were more effective than the administration of IV fluids alone for the prevention of intra-operative fluid overload requiring subsequent endotracheal reintubation during the post-operative period. Their studies confirmed our findings but neither Jiang nor Ponnudurai, *et al*, considered renal dysfunction after liver transplantation [23, 24]. 

Renal dysfunction is very common after liver transplantation. Pre-operative renal dysfunction and numerous intra-operative factors such as cross-clamping of the vena cava with decreased renal perfusion pressure, decreased intravascular volume, and systemic hypotension can contribute to post-operative renal dysfunction [12]. A study conducted by Schroeder, *et al* [15], showed increased morbidity and even mortality in patients with multisystem organ impairment who were deliberately kept hypovolemic that compromised perfusion of their vital organs. Therefore, if patients with ESLD and potentially impaired renal function are kept hypovolemic in an effort to decrease hemorrhage during surgery, their morbidity and mortality would be increased. In the present study, the central venous pressure was kept in the range of 8–10 cm H_2_O in the restricted crystalloid fluid group; the patients also received vasopressor to increase the splanchnic arterial tone and renal blood flow in order to prevent renal dysfunction. 

This study had some limitations. We should have followed respiratory insufficiency with other markers such as p_a_O_2_/p_A_O_2_ and renal function by other markers, such as neutrophil gelatinase-associated lipocalin, and glomerular filtration rate**.** Therefore, more studies are recommended to follow patients with these markers.

According to present study, during the OLT for ESLD, controlled administration of restricted crystalloid fluid prevents early post-operative renal insufficiency and respiratory insufficiency.
